# Differential regulation of the zebrafish *orthopedia1 *gene during fate determination of diencephalic neurons

**DOI:** 10.1186/1471-213X-6-50

**Published:** 2006-10-30

**Authors:** Luca Del Giacco, Paolo Sordino, Anna Pistocchi, Nikos Andreakis, Raffaella Tarallo, Barbara Di Benedetto, Franco Cotelli

**Affiliations:** 1 Department of Biology, Università degli Studi di Milano, Via Celoria 26, Milano, 20133, Italy; 2 Laboratory of Biochemistry and Molecular Biology, Stazione Zoologica "A. Dohrn", Villa Comunale, Napoli, 80121, Italy; 3 GSF National Research Center for Environment and Health, Institute of Developmental Genetics, Ingolstaedter Landstrasse 1, 85764 Neuherberg, Germany

## Abstract

**Background:**

The homeodomain transcription factor Orthopedia (Otp) is essential in restricting the fate of multiple classes of secreting neurons in the neuroendocrine hypothalamus of vertebrates. However, there is little information on the intercellular factors that regulate *Otp *expression during development.

**Results:**

Here, we identified two *otp *orthologues in zebrafish (*otp1 *and *otp2*) and explored *otp1 *in the context of the morphogenetic pathways that specify neuroectodermal regions. During forebrain development, *otp1 *is expressed in anterior groups of diencephalic cells, positioned in the preoptic area (PO) (anterior alar plate) and the posterior tuberculum (PT) (posterior basal plate). The latter structure is characterized by Tyrosine Hydroxylase (TH)-positive cells, suggesting a role for *otp1 *in the lineage restriction of catecholaminergic (CA) neurons. Disruptions of Hedgehog (HH) and Fibroblast Growth Factor (FGF) pathways point to the ability of SHH protein to trigger *otp1 *expression in PO presumptive neuroblasts, with the attenuating effect of Dzip1 and FGF8. In addition, our data disclose *otp1 *as a determinant of CA neurons in the PT, where *otp1 *activity is strictly dependent on Nodal signaling and it is not responsive to SHH and FGF.

**Conclusion:**

In this study, we pinpoint the evolutionary importance of *otp1 *transcription factor in cell states of the diencephalon anlage and early neuronal progenitors. Furthermore, our data indicate that morphogenetic mechanisms differentially regulate *otp1 *expression in alar and basal plates.

## Background

The neurosecretory system controls a wide variety of behavioural processes through synthesis and release of different neurotransmitters in the peripheral and central nervous systems [1,2]. One of the key integrating centers of this organization is the hypothalamus, located in the ventral sector of the diencephalon. However, insights in the development of endocrine neurons has been gained in the midbrain, due to implications with mental and neurological phenotypes that involve growth, reproduction and general homeostasis, and have clinical relevance in degenerative and psychiatric disorders of embryonic origin (*e.g*. Parkinson's disease and addiction) [3-8]. A wealth of genetic data shows that the combinatorial codes of early instructional cues from Hedgehog (HH), Fibroblast Growth Factor (FGF), and Transforming Growth Factor (TGF-β) extracellular signals mediate differentiation of dopaminergic (DA) neurons in the midbrain [6,9-15]. Knowledge of the mechanisms of action connecting the prosencephalic signaling pathways, the expression of specific transcription factors and the specification of neuronal individuality during the development of the hypothalamus remains largely unclear [15].

Because of its central contribution to hypothalamic phenotypes during ontogenesis, the homeobox-containing *orthopedia *(*otp*) gene may allow a meaningful understanding of signaling cascades [16,17]. Homologs of *otp *have been identified in almost all Metazoa, pointing to a conservative and fundamental role in patterning and differentiation [18-28]. In mouse forebrain, *Otp *is expressed in anterior and posteroventral hypothalamus, retrochiasmatic and supraoptic/paraventricular areas [18]. *Otp *knock-out mice embryos fail to properly differentiate anterior periventricular, paraventricular and supraoptic nuclei, responsible for secretion of somatostatin, arginine vasopressin, oxytocin, corticotropin-releasing hormone and thyrotropin-releasing hormones [16,17]. In the diencephalon, Otp acts in parallel with the bHLH-PAS domain factor Sim1 and its dimerizing partner ARNT2, and triggers expression of *sim2 *and *brn2*, a POU domain factor [16,17,29-32]. It has been shown that *otp *is a direct target of Brachyury and Spdeadringer transcription factors in vertebrates and invertebrates [33,34]. Recently, several transcription factors have been proposed as candidates for upstream regulatory interactions with echinoderm *otp *[35]. Some cases of holoprosencephaly, a congenital disorder with deficiencies in specific groups of CA neurons, are characterized by disruption of *sonic hedgehog *(*shh*) and secondary down-regulation of *otp*, *brn2 *and *sim1*, evoking an interaction between *otp *and *shh *[36]. Despite all these evidences, the mechanisms of regulation of the *Otp *gene itself remain largely obscure.

We took advantage of the zebrafish to investigate the *in vivo *functions of *shh*, *fgf8*, and *ndr2 *signaling pathways on *otp1 *expression by means of morpholino-, mRNA- and mutant-based methodologies. We provide evidence that SHH regulates *otp1 *neuronal differentiation in the rostral preoptic area (PO) (anterior alar diencephalon), through the antagonistic interaction with FGF and Dzip1, a zinc-finger/coiled-coil domain protein [37-40]. Moreover, we also demonstrate that SHH- and FGF8-independent, Ndr2-dependent transcription of *otp1 *in the posterior tuberculum (PT) (posterior basal diencephalon) is necessary to trigger CA neuronal fates. In evolutionary perspectives, functional association between *otp1 *gene expression and patterning of the diencephalon embodies a primitive condition in the rise of neuronal complexity of vertebrates.

## Results

### Isolation and structural analysis of the zebrafish orthopedia genes

**Figure 1:**
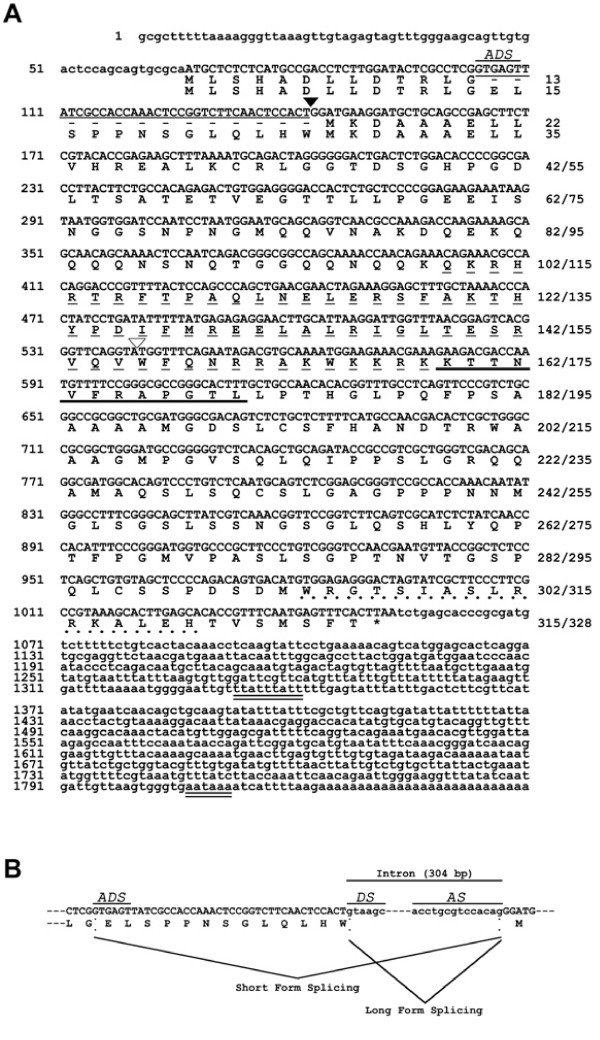
***Organization and transcriptional mechanisms of the zebrafish otp1 gene***. ***A***, *otp1 *mRNA is alternatively spliced and encodes two forms of the protein. On the right are shown the amino acid residues of the short and the long form of Otp1, respectively. The solid arrowhead indicates the position of the 304-nucleotide (nt) intron involved in the alternative splicing. The deleted 39-nt region of the *otp1 *mRNA resulting in the short form of the protein is shown by a thin solid line. The additional donor site triggering the 39-nt deletion is also indicated (ADS). The open arrowhead points to the position of the second intron. The asterisk indicates the stop codon at the end of the ORF. The *AATAAA *polyadenylation site and the *TTATTTATT *motif that mediates mRNA degradation are double-underlined. The thin dashed line marks the homeodomain, the thick solid line marks the conserved 12 amino acids downstream of the homeodomain and the bold dotted line marks the OAR domain. ***B***, Alternative splicing pattern of the *otp1 *nuclear RNA. The sequences of the donor (DS) and the acceptor sites (AS) of the 304-nt intron are shown, together with the flanking regions of the coding sequence. The alternative donor site (ADS) located in the upstream exon that determines the deletion of the 39-nt region (solid line) is also indicated. The sequence has been submitted to the GenBank/EMBL database under accession number AF071496.

To isolate the zebrafish homologue of *orthopedia *(*otp1*), a 16–40 hour old zebrafish embryo cDNA library (kindly provided by P. Chambon) was used for a PCR-based screening with degenerate primers. Primers, named ort1_fw (5'-CCNGCNCAGCTSAACGA-3'), and ort2_rv (5'-CKYTTYTTCCAYTTNGC-3'), correspond to PAQLNE and AKWKKR regions of the mouse Otp homeodomain, respectively. Using a semi-nested PCR approach, we isolated a cDNA band of 150 base pairs (bp) encoding a partial homeodomain identical to the corresponding residues in the mouse Otp protein. Gene- and vector-specific primers were used on the same library to isolate the 5' and 3' ends of the mRNA, allowing the identification of a 1823 bp (poly(A) tail excluded) full-length *otp1 *cDNA containing a 987 bp-long open reading frame (Fig. 1A). The 5' UTR of the mRNA is 66-nucleotide (nt) long and the first putative AUG codon is located between nt 67 and 69, suggesting the 67/69 AUG triplet as the initiation codon. Therefore, the deduced protein encoded by *otp1 *is 328 amino acid (aa) long. The mRNA has a UAA stop translation codon at nt 1051 and a 770 nt long 3' UTR region, with one polyadenylation signal (AATAAA) located between nucleotides 1807 and 1812.

**Figure 3:**
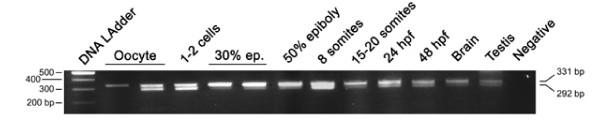
***Developmental RT-PCR analysis of the otp1 transcripts***. Ethidium Bromide-stained agarose gel of RT-PCRs performed using the z*otp*S and z*otp*Rv primers: DNA Ladder (lane 1), oocyte (lanes 2 and 3), 1–2 cell stage (lane 4), 30% epiboly (lanes 5 and 6), 50% epiboly (lane 7), 8 somite stage (lane 8), 15–20 somite stage (lane 9), 24 hpf (lane 10), 48 hpf (lane 11), brain (lane 12), testis (lane 13), and negative control (lane 14) in absence of cDNA. 80% epiboly, 1–2 somites, and 10 somites samples (not shown) resulted positive after the analysis. On the left of the panel is shown the size of the DNA Ladder bands, on the right is indicated the size of the 2 PCR products, 292 bp and 331 bp, corresponding to the short and the long form of the *otp1 *mRNA, respectively.

During the same screening, a shorter *otp1 *transcript was also identified that encodes a 315 aa long form of *otp1*. The two transcripts differ for a 39-nt insertion at 5' end. Interestingly, both human and mouse *otp *genes have an intron at the same position of the zebrafish *otp1 *insertion, suggesting that the two isoforms are generated by alternative splicing. Analysis of the zebrafish *otp1 *genomic region flanking the insertion confirmed the presence of an intron (located between nucleotides 142 and 143 of the longer transcript) at the same position as in human and mouse *Otp *genes (Fig. 1A). In zebrafish, an additional donor sites generates a shorter mRNA form when a 39-nt fragment at the 3' end of the first exon is spliced out together with the downstream intron (Fig. 1B). RT-PCR experiments and a cDNA library-based PCR screening confirmed the existence of two *otp1 *transcripts during embryo development (Fig. 3). Genomic analysis of the zebrafish *otp1 *gene, located bioinformatically on chromosome 15, has been confirmed by PCR, revealing an organization similar to mammalian *otp *homologs. The gene contains two introns of 304 and 1144 bp, with the first one interrupting the N-terminus of the protein, and the second one located at the very end of the homeobox.

*otp1 *mRNA is characterized by an AU-rich element (ARE) of ~150 nt in length, that lies within the 3' UTR and is located ~200 bases downstream of the stop codon. This ARE contains five copies of the AUUUA sequence, one of which overlaps with the UUAUUUAUU nonamer, that represent the minimal AU-rich motif that is able to destabilize and decay messengers [41].

*In silico *search of the zebrafish genome using *otp1 *cDNA as bait provided a second *otp *gene, named *otp2*, located on chromosome 2. *otp2 *ORF is 999 bp long, corresponding to a putative protein of 332 aa. According to mRNA sequences submitted to the NCBI database (accession nos. XM_678094 and XM_701814), the *otp2 *RNA is alternatively spliced and encodes two discrete forms of mature mRNA. Genomic organization of *otp2 *is identical to *otp1*, including presence and position of introns and the additional donor site (data not shown).

### Molecular evolution of OTP proteins

Zebrafish Otp1 and Otp2 proteins clearly belong to the Otp protein family of transcription factors, and they are likely co-orthologs arisen from the ancient genome duplication event that occurred early in the phylogeny of ray-finned fish [42-45]. The identity of zebrafish Otp homeodomains with *Mus musculus*, *Dugesia japonica *and *Drosophila melanogaster *is 100%, 97% and 95%, while full protein identity with mouse is 78% for Otp1 and 81% for Otp2. In addition, they appear to share the Otp-specific transactivating regions, as well as two additional domains near the terminator codon [18] (Fig. 1A). The 13 aa stretch resulting from alternative splicing (see above) of Otp proteins is characteristic of the fish lineage (see above). In fact, mouse and human *Otp *genes lack the additional donor site in the first intron responsible for the alternative splicing of the long protein form. For this reason, the short form of the zebrafish Otp proteins shows higher levels of similarity with mammalian homologs (data not shown).

**Figure 2:**
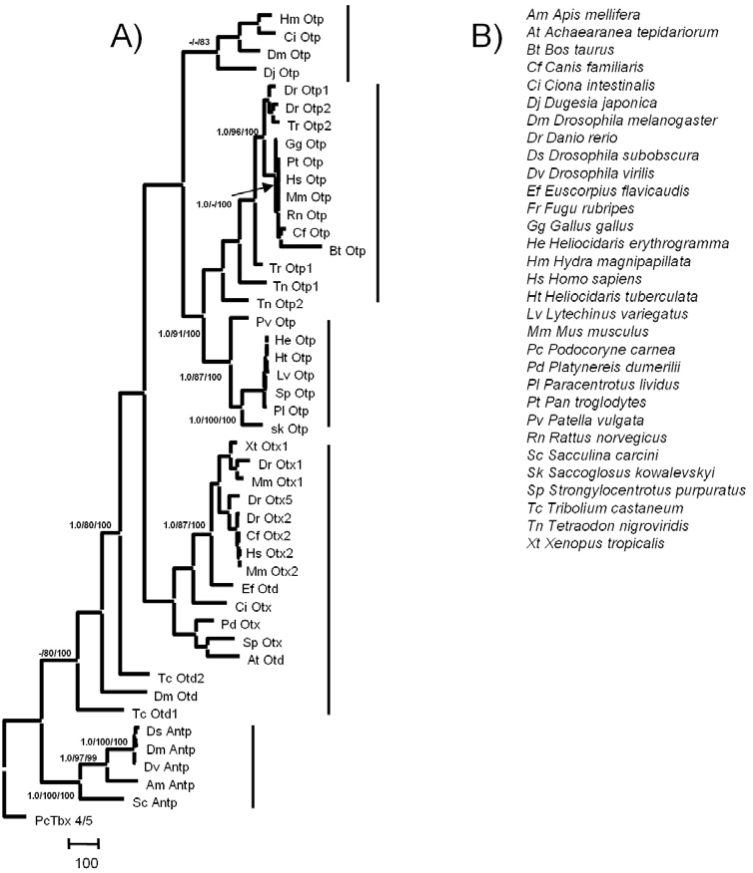
***Phylogenetic relationship of the Otp gene family***. ***A***, MP phylogenetic reconstruction of Otp, Otd, Otx, and Antp protein sequences. BPP/MP/NJ: Bayesian posterior probability, Maximum Parsimony, Neighbour Joining bootstrap support. A dash denotes BPP values below 95% and bootstrap values below 80%. *Podocoryne carnea *Tbx4/5 protein was used as an outgroup. ***B***, List of taxa with abbreviations (see *Methods *for accession numbers).

For phylogenetic analysis, we used all available Otp protein and nucleotide sequences, including four ones that were derived from EST and genomic sequence databases (*Hydra magnipapillata*, *Oryzias latipes*, *Takifugu rubripes *and *Tetraodon nigroviridis*). Otp protein alignment consisted of 591 characters, 408 of which were parsimony-informative. Zebrafish Otp sequences were easily aligned against the rest of the data set mainly within the homeobox domain, including small secondary regions of high similarity. Only these regions were used for the sequence analysis, while the most variable fragments were excluded. The Maximum Parsimony (MP) phylogenetic reconstruction (Fig. 2) resulted in 300 equally parsimonious trees (4458 steps of length; consistency index = 0.73; retention index = 0.76). Maximum likelihood (ML) and Bayesian phylogenetic analyses (data not shown), constrained with the model of protein evolution that fits the data best, (JTT; proportion of invariable sites; Gamma distribution, shape parameter α = 2.318) [46] were topologically similar to the MP tree. At any phylogenetic reconstruction, the Antennapedia homeodomain region diverges greatly from the other gene clusters, occupying a distinct basal position. Otp, Otd and Otx gene families share an elevated sequence and structural homology in their homeodomain region. Within the Otp gene-family, two sub-clades are highly supported by all phylogenetic reconstruction methods: a vertebrate-specific Otp protein cluster, including fish paralogs, and one including Mollusca, Echinoderma and Hemichordata orthologs. A third sub-clade consisting of *D. japonica*, *D. melanogaster*, *H. magnipapillata*, and *Ciona intestinalis *Otp gene sequences is not robustly supported in any of the topologies (Fig. 2). Finally, a Otd/Otx gene cluster, distantly related and basal to the Otp gene family, was observed in all phylogenies inferred.

### Spatiotemporal expression of otp1 during embryogenesis

#### RT-PCR

We evaluated the presence of the two mRNA forms at different stages by means of RT-PCR using a pair of primers spanning the cDNA region involved in alternative splicing. Both transcripts are already present at the oocyte stage, proving the maternal origin of the transcript, and remain detectable throughout the analyzed stages (Fig. 3). We also report the expression of *otp1 *in the adult fish, showing for the first time the activity of the gene in the adult brain and in non-neuroectodermal territories (testis). The RT-PCR approach does not recognize trends or relative differences between mRNA forms and developmental stages, supporting the view that splicing of *otp1 *RNA is not subject to strict regulatory mechanisms (Fig. 3).

#### Hindbrain

**Figure 4:**
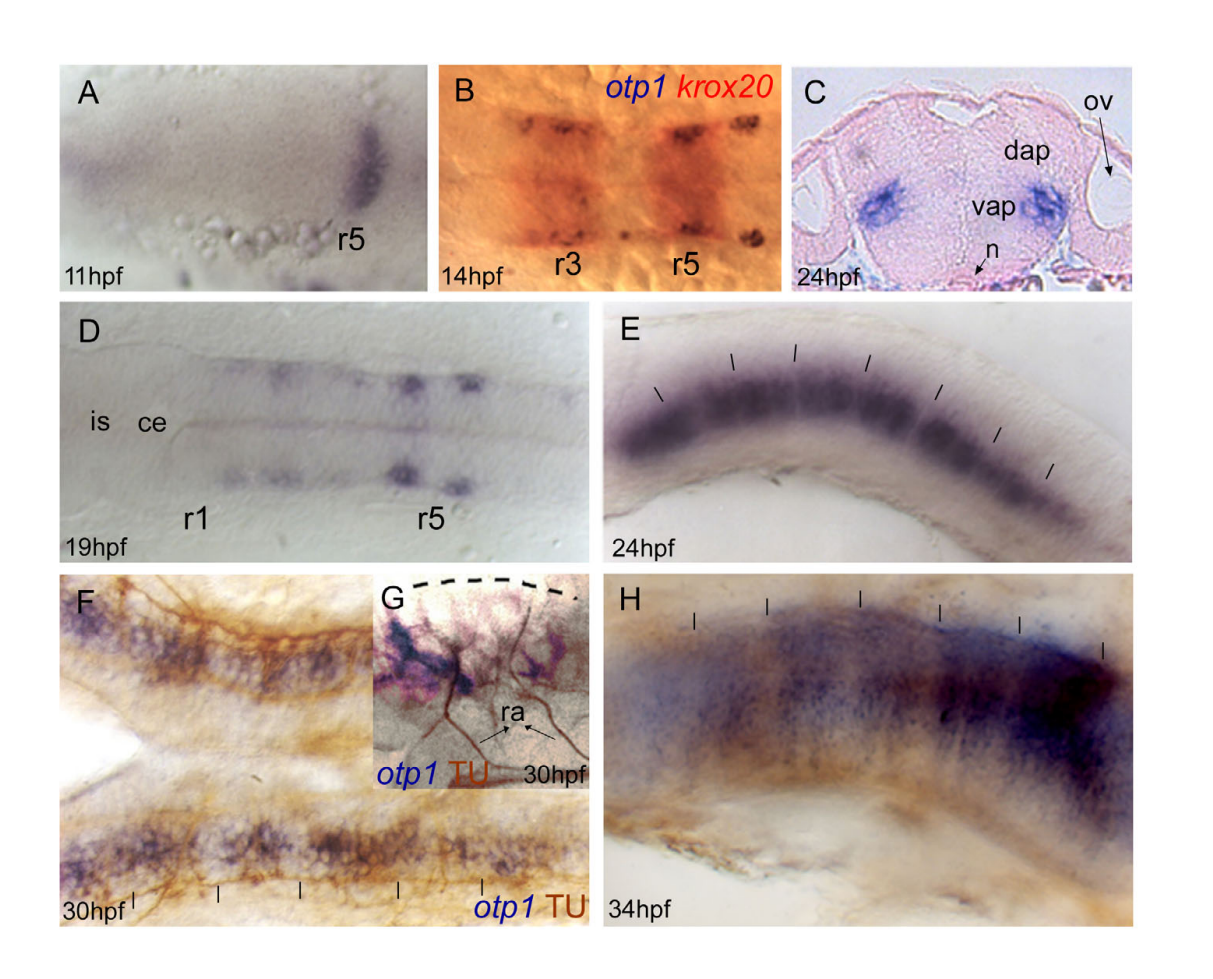
***Zebrafish otp1 expression in the hindbrain***. Anterior is left in all images except for ***C***. All views are dorsal except for ***E***, ***G ***and ***H ***that are lateral. ***A***, At 11 hpf stage, a narrow transverse stripe encompasses the neural tube in its anterior half. During somitogenesis, a second narrow stripe appears more anteriorly (data not shown). ***B***, Double labelling with *otp1 *(blue) and *krox20 *(red) shows that *otp1 *expression in the hindbrain labels rhombomere 3 (r3) and 5 (r5) at 14 hpf stage. This developmental window coincides with the restriction of the two *otp1 *stripes toward the lateral margins of the neural tube, and with the early extension of the signal throughout the rhombencephalon. ***C***, Transverse section of a 24 hpf stage zebrafish embryo through the hindbrain shows that these lateral clusters belong to the mantle layer of the ventral alar plate and are placed medially along the dorsoventral axis. ***D***, Following (19 hpf), *otp1 *signals extend anteriorly, and then posteriorly, in the shape of repeated patterns. ***E***, 24 hpf embryo; *otp1 *mRNA signal localizes in restricted groups of cells, in a pattern reminiscent of rhombomere segmentation. ***F***, ***G***, *otp1 *WISH combined with acetylated α-tubulin (TU) immunochemical staining confirms that *otp1 *transcripts occur in paired clusters within each rhombomere (30 hpf) (open arrowheads in ***F ***indicate the axons in the central part of every rhombomere). ***H***, At 34 hpf, *otp1 *domains have elongated dorsally in parallel columns (vertical dashes in ***E***, ***F ***and ***H***, and dashed line in ***G***, indicate rhombomere boundaries and profile, respectively). ce, cerebellum; dap, dorsal alar plate; is, isthmus; n, notochord; ov, otic vesicle; ra, reticulospinal axons; vap, ventral alar plate.

The spatial and temporal distribution of *otp1 *transcripts was examined by whole mount *in situ *hybridization (WISH) following standard protocols with digoxigenin and fluorescein-UTP-labeled probes [47]. Although RT-PCR revealed the presence of maternal and zygotic transcripts, first evidence of *otp1 *mRNA localization by WISH appears in the forming hindbrain around the 3 somites (s) stage, as a transverse stripe in the prospective rhombencephalon (Fig. 4A). As shown by double labelling with *krox20*, *otp1 *expression at 12 s occurs in rhombomeres 3 (r3) and 5 (r5) (Fig. 4B). A histological cross section at the level of the otic placodes shows that rhombencephalic *otp1 *cells are located in the ventral alar plate of the neural tube, within the mantler layer (Fig. 4C). As somitogenesis proceeds, *otp1 *expression extends rostrally (Fig. 4D) and then caudally across the hindbrain in the shape of segmentally iterated groups of cells (Fig. 4E). At 34 hours post fertilization (hpf), *otp1 *transcripts in the hindbrain extend dorsally as parallel stripes in the middle of each neuromeric segment (rhombomere), possibly acting as guidance cues for axonal elongation (Fig. 4H). This hypothesis is supported by spatial comparison of *otp1 *mRNA with acetylated α-tubulin at 30 hpf, a marker of reticulospinal neurons and corresponding axons in the central part of every rhombomere [48] (Fig. 4F,G). At 48 hpf, *otp1 *expression in the hindbrain still persists in every rhombomere without further modifications (data not shown).

#### Diencephalon

**Figure 5:**
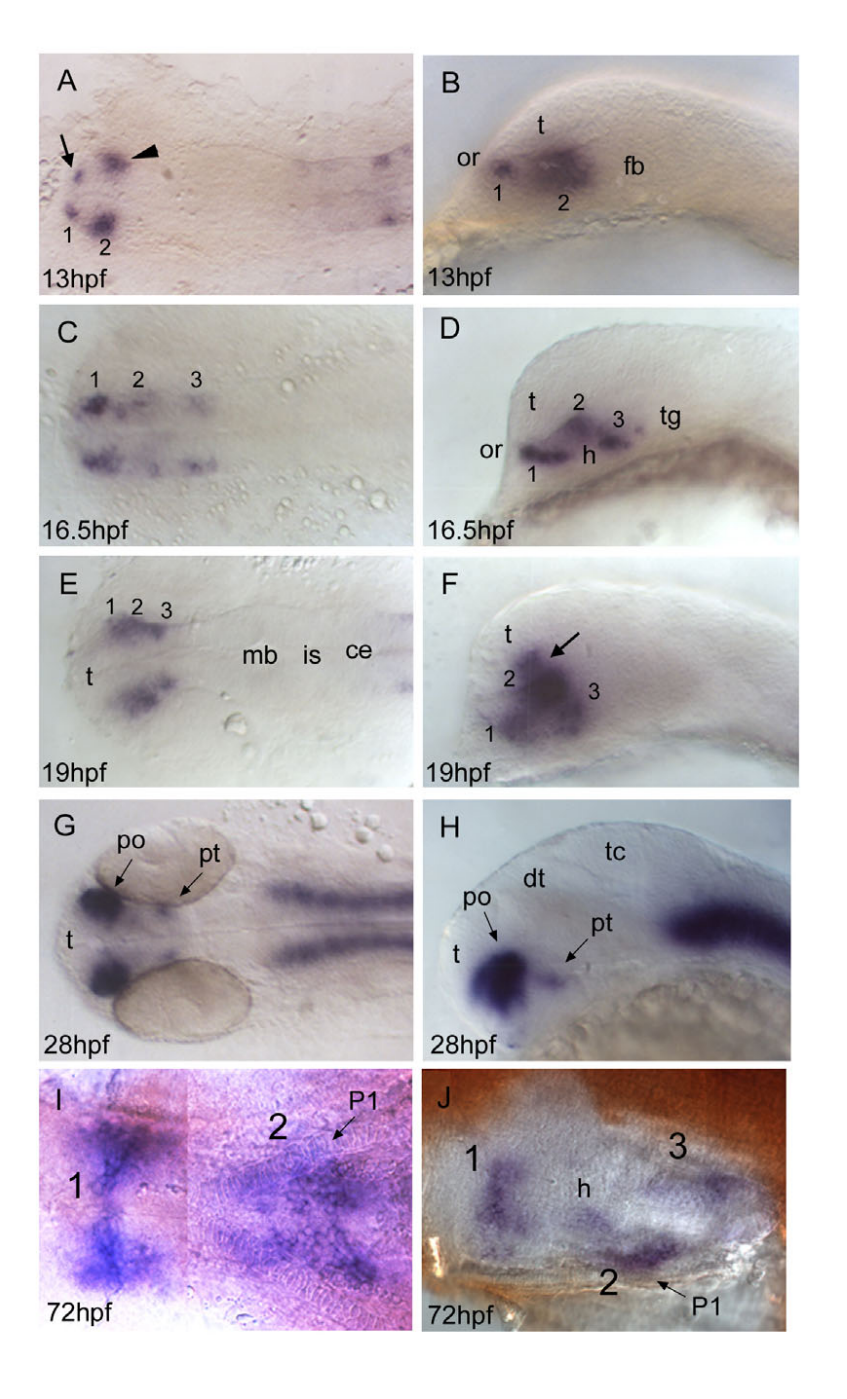
***otp1 expression in the forebrain***. Anterior is always to the left. Except ***I***, a ventral view, right panels are lateral views and left ones are dorsal. Eyes have been removed in ***I ***and ***J***. ***A***, ***B***, About 1 h after early *otp1 *expression in the hindbrain (11 hpf, see Fig. 4A), a small lateral domain appears at the anterior edge of the central nervous system (arrow) (see also Fig. 6A). Shortly after, a novel center of transcription emerges posterolaterally in the alar plate (arrowhead). ***C***, ***D***, Then, *otp1 *expression diffuses posteriorly in a segmented fashion with three clusters (1–3) (16.5 hpf). During this developmental phase, spatial rearrangements of the anterior forebrain start occurring as the result of the ventral bending of the neuraxis [50]. Even though intrinsic topological changes in *otp1 *transcription may also happen, their extents are eventually obscured by morphogenetic dislocations in the neural tube. ***E***, ***F***, At 19 hpf, *otp1 *positive cells in the middle of longitudinal domains displace dorsally with respect to cells at different axial positions. Consequently, *otp1 *labelling in the anterior forebrain coalesces in the proximity of the optic recess (arrow). ***G***, ***H***, 28 hpf WISH with a large compacted anterior diencephalic cluster in PO and a small one caudally in PT. ***I***, ***J***, Later in development (72 hpf), *otp1 *expression is detected in three distinct domains (1–3) in the ventral diencephalon. Unlike early in development, these three clusters are fused along the midline. ce, cerebellum; dt, dorsal thalamus; fb, forebrain; h, hypothalamus; is, isthmus; mb, midbrain; P1, pharyngeal arch 1; po, preoptic area; pt, posterior tuberculum; t, telencephalon; tc, tectum; tg, tegmentum.

**Figure 6:**
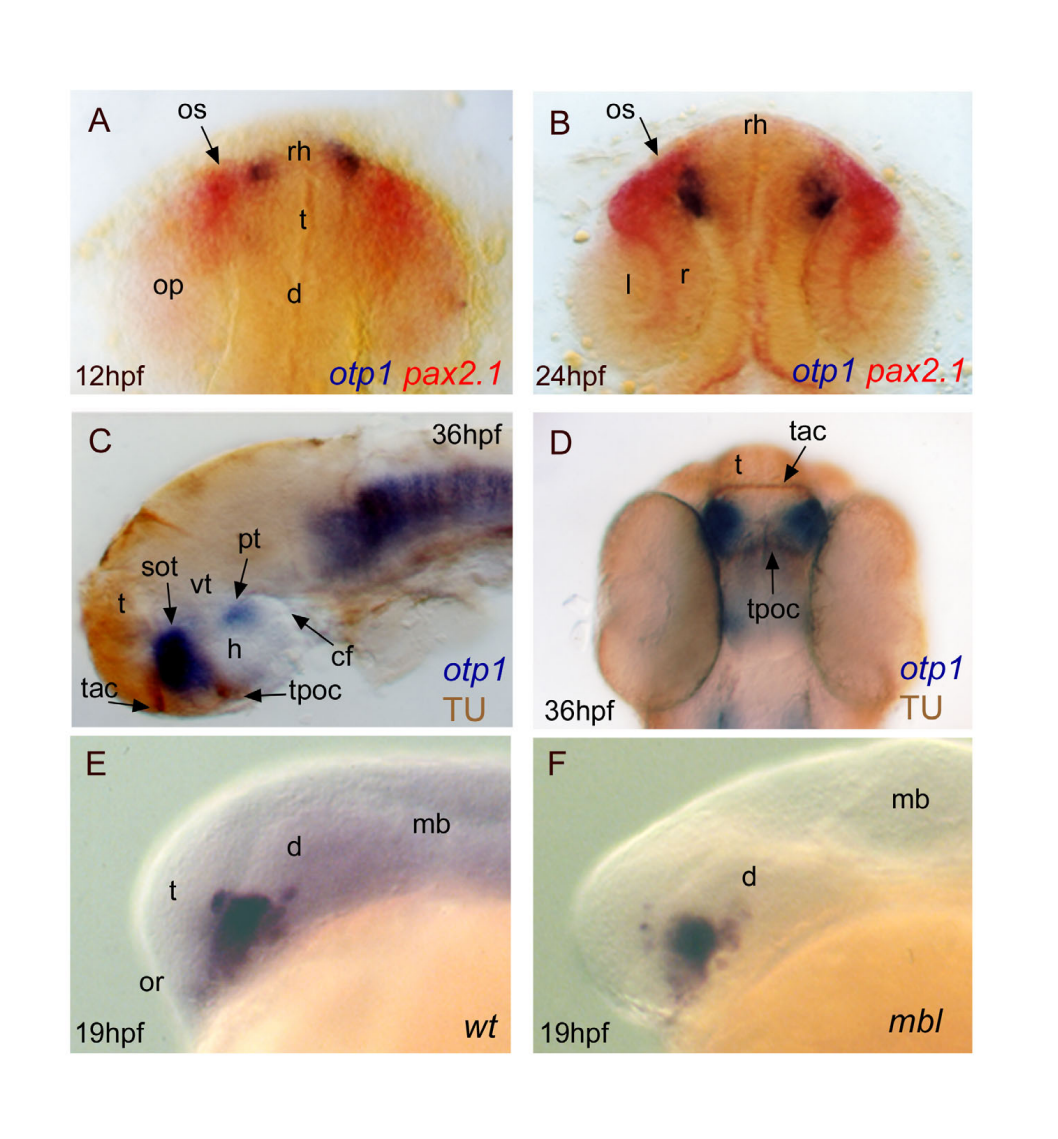
***Topology of otp1 transcripts in forebrain compartments***. Anterior is top in ***A***, ***B ***and ***D ***dorsal views, otherwise left and lateral view panels. Eyes have been removed in ***C***. ***A ***(12 hpf), ***B ***(24 hpf), early *otp1 *expression foci (blue) occur at the anteromedial edge of *pax2.1 *(red) pattern in the optic stalks, as shown by double WISH. ***C***, ***D***, Co-labelling with anti-acetylated α-tubulin antibody (brown) and *otp1 *riboprobe (blue) demonstrates that the anterior *otp1 *cell cluster is delimited by the supraoptic tracts and those of the anterior and posterior commissures (36 hpf). ***E***, ***F***, *mbl *embryos are not altered in the dorsal extension of the PO *otp1 *cluster, if compared with control embryos (19 hpf). cf, cephalic flexure; d, diencephalon; h, hypothalamus; l, lens; mb, midbrain; op, optic primordia; or, optic recess; os, optic stalks; r, retina; rh, rostral hypothalamus; sot, supraoptic tract; t, telencephalon; tac, tract of the anterior commissure; tpoc, tract of the postoptic commissure; vt, ventral thalamus.

Soon after labelling in the hindbrain, a distinct cluster of *otp1 *transcription appears at the rostral end of the neural tube, in medial position with respect to the optic stalk-specific *pax-2.1 *positive cells [49] (Fig. 6A,B). Shortly after, a novel diencephalic domain emerges posteriorly to the previous one (Fig. 5A,B). The foremost, smaller group is located at the boundary with the prospective telencephalon, while the posterior, larger one is observed more laterally, below the optic recess (Fig. 5A,B). At 16.5 hpf, *otp1 *expression in the hypothalamus has gained further spatial complexity, as shown by the evidence of three main clusters (Fig. 5C,D). The 16 s stage marks the onset of ventral bending of the neural axis at the level of the cephalic flexure, a major morphogenetic process that generates the displacement of forebrain territories at distinct axial levels [50]. In this framework, a spatial rearrangement of the hypothalamic *otp1 *expression is observed. Apparently, the medial cluster of *otp1 *expression is displaced dorsally and merges with the anterior one (compare clusters 1–3 in Fig. 5B,D,F). At 24 hpf, *otp1 *expression in the hypothalamus is established as two domains, one in the anterior alar plate, at the boundary with the telencephalon (areas termed d1 and d2 [51]), and one in the posterior basal plate of the hypothalamus, in few cells of the posterior tuberculum (PT) extending from the hairpin bend near the cephalic flexure (areas d5 and d6 [51]) (Fig. 5G,H). At 72 hpf, again three domains of *otp1 *mRNA distribution are detected in the ventral diencephalon (Fig. 5I,J).

At 24 hpf, transversal histological sections show that the anterior diencephalic domain belongs to the non-germinal (mantler) layer (data not shown). Coupling *otp1 *WISH with acetylated α-tubulin immunostaining, a marker of major axonal traits in the embryonic CNS, proves that the anterior *otp1 *cluster is enclosed in the preoptic area (PO) (alar plate) by the tracts of the anterior and postoptic commissures, and it extends toward the trait of the supraoptic commissure (Fig. 6C,D). Due to this dorsal extension in the supraoptic region, we addressed *otp1 *expression at the telencephalic-diencephalic boundary using *masterblind *(*mbl*) mutants. In *mbl *embryos, several forebrain defects are observed, such as absence of optic vesicles, olfactory placodes, clusters of primary telencephalic neurons, anterior and postoptic commissures [52-54]. Despite profound alterations in the telencephalic structures, no significant changes are detected in the PO cluster of *otp1 *expressing cells in comparison with *wt *embryos at 20-s stage, suggesting that *otp1 *expression does not occur in the telencephalon (Fig. 6E,F).

### Combinatorial regulation of otp1 by Hedgehog, Fibroblast Growth Factor and Nodal signaling

**Figure 7:**
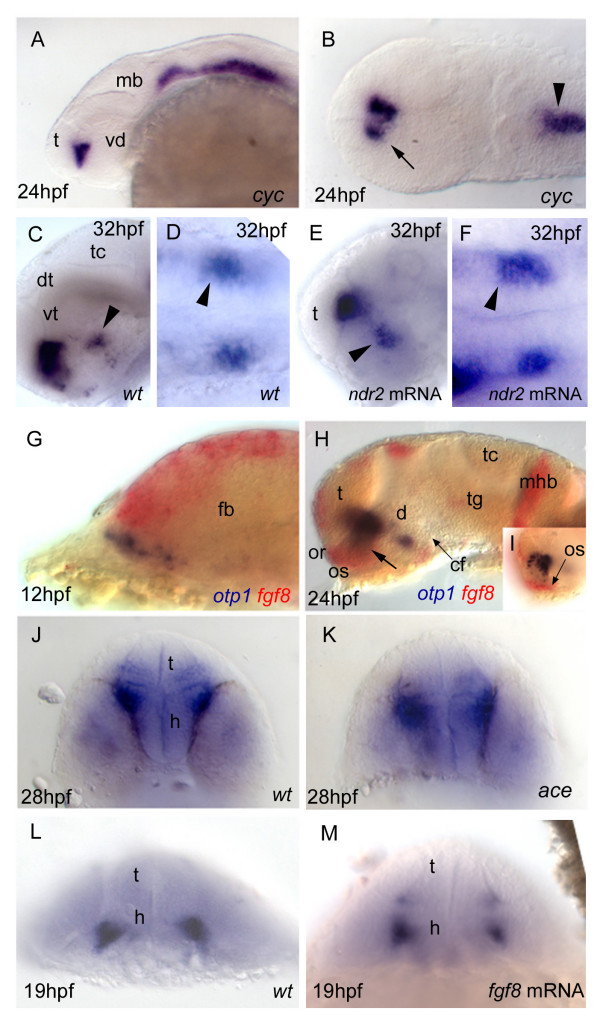
***Nodal and FGF signalings are related with otp1 in the diencephalon***. Anterior is left in all but ***J***-***M ***panels. ***J***-***M ***panels are frontal views, ***B***, ***D ***and ***F ***are dorsal, and the remaining ones are lateral. ***A***, ***B***, Nodal signals emanating from mesendoderm and overlaying neuroectoderm represent one of the most important inducing pathways that govern regional patterning of the neural tube. Zebrafish *cyclops *(*cyc*) mutant embryos lack a functional floor plate, and indirectly disrupt HH signaling [55, 56, 57, 58]. At 24 hpf, *cyc *mutants display complete loss of *otp1 *expression in the PT, and show an asymmetric reduction of PO domains (arrow). Forebrain and hindbrain patterns are closer or fused along the midline (arrowhead). ***C***-***F***, Accordingly, the essential role of Nodal signaling in *otp1 *transcription in the posterior hypothalamus is confirmed by microinjection of *ndr2 *mRNA whereas, compared with controls (***C***, ***D***), *otp1 *domain in the PT is expanded (***E***, ***F***) (arrowhead in ***C***-***F ***indicates the PT-specific *otp1 *expression) (32 hpf). ***G***-***I***, Double WISH reveals that *fgf8 *(red) and *otp1 *(blue) expression patterns are complementary at the boundary between prospective telencephalon and diencephalon since early somitogenesis stages (***G***, 5 s).***H***, At 24 hpf, PO *otp1 *expression is adjacent to *fgf8*-positive optic stalks (arrow). ***I***, A high power image of ***H ***illustrates the physical proximity of *otp1 *and *fgf8 *transcripts. ***J***, ***K***, *fgf8 *gene activity is abolished in *acerebellar *(*ace*) mutant [37]. Examination of an optic section of *ace *embryos at 28 hpf through the optic area shows that the PO-specific *otp1 *cluster is expanded radially (***K***) in comparison with controls (***J***). ***L***, ***M***, Overexpression of *fgf8 *induces a reduction of the number of *otp1 *expressing cells in the PO, whereas this cluster also fails to properly coalesce, likely due to developmental delay (19 hpf). On the contrary, the PT-specific *otp1 *domain is unaltered (data not shown). cf, cephalic flexure; d, diencephalon; dt, dorsal thalamus; fb, forebrain; h, hypothalamus; mb, midbrain; mhb, midbrain-hindbrain boundary; or, optic recess; os, optic stalks; t, telencephalon; tc, tectum; tg, tegmentum; vd, ventral diencephalon; vt, ventral thalamus.

The *cyclops *(*cyc*) embryos carry mutations in the *nodal-related 2 *(*ndr2*) gene that alter the development of ventral CNS structures, with a severe phenotype in the rostral brain [55-58]. Analysis of *otp1 *expression in 24 hpf *cyc *embryos with intermediate phenotypes (reduction of the ventral diencephalon and partial fusion of the eyes) shows a reduced size and midline blending of the PO domain. Interestingly, no *otp1 *expression occurs in the PT of *cyc *embryos (Fig. 7A,B). To answer whether this result is determined by impaired development of the *otp1*-expressing cells or by absence of the prosencephalic ventral structures where the *otp1*-positive neurons normally form, we microinjected *ndr2 *synthetic mRNA and analyzed the effects on the expression profile of *otp1 *in the PT area (Fig. 7C–F). In accordance with the absence of *otp1 *signal in *cyc *embryos with intermediate phenotypes, *ndr2 *overexpression induces a moderate expansion of the PT-specific *otp1 *domain (up to 25% of the injected embryos) (compare Fig. 7C with 7E, and Fig. 7D with 7F).

**Figure 9:**
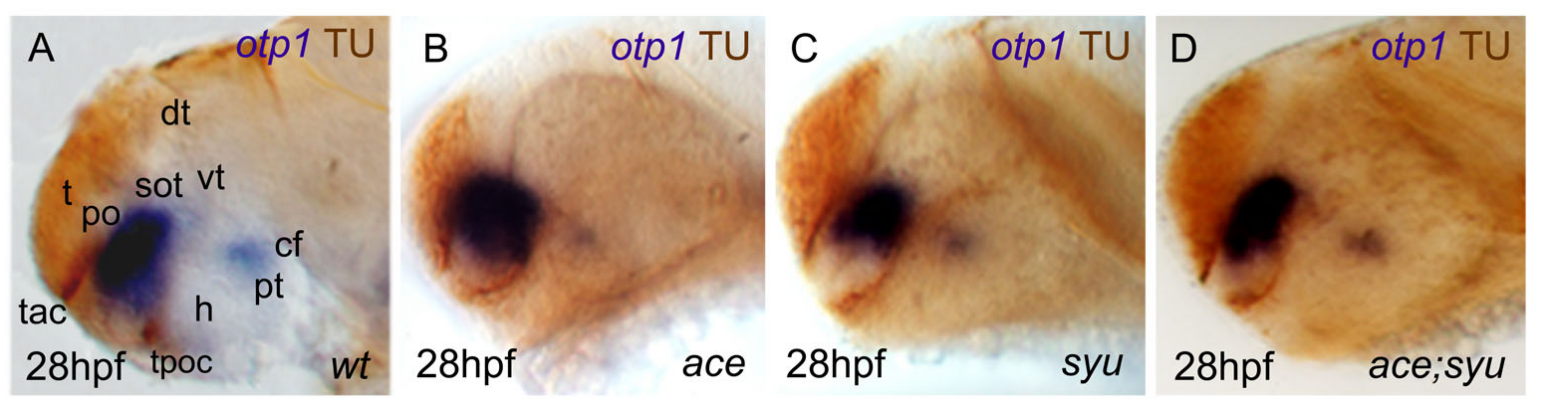
***Synergicistic effects of HH and FGF signaling on otp1 expression in the PO cluster***. Anterior is left and dorsal is up in all panels. Eyes have been removed in ***A***. All embryos are 28 hpf and have been double stained with *otp1 *riboprobe and anti-acetylated α-tubulin (TU) antibody. Compared with the *wild-type *PO (***A***), the anterior *otp1 *cluster is expanded and reduced, respectively, in *ace *(***B***) and *syu *(***C***), but its normal size is rescued in double *ace*; *syu *mutant embryos (***D***). cf, cephalic flexure; dt, dorsal thalamus; h, hypothalamus; po, preoptic area; pt, posterior tuberculum; sot, supraoptic tract; t, telencephalon; tac, tract of the anterior commissure; tpoc, tract of the postoptic commissure; vt, ventral thalamus.

FGFs released from the isthmus and other organizing centres are required to pattern the neuroepithelium along its dorsoventral and anteroposterior axes [59,60]. In zebrafish embryo, tissues expressing *fgf8 *are observed in close proximity with the *otp1 *cluster in PO (Fig. 7G–I). Herein, the specification of *otp1 *cells in the PO is repressed in 13 to 24% of the embryos at 19 hpf that overexpress FGF8 (Fig. 7L,M). The same cluster is expanded in *fgf8 *(*acerebellar*, *ace*) mutant embryos at 28 hpf (Fig. 9A,B). On the contrary, PT-specific *otp1 *expression is not altered in both *ace *embryos and following *fgf8 *overexpression (Fig. 9B; data not shown).

**Figure 8:**
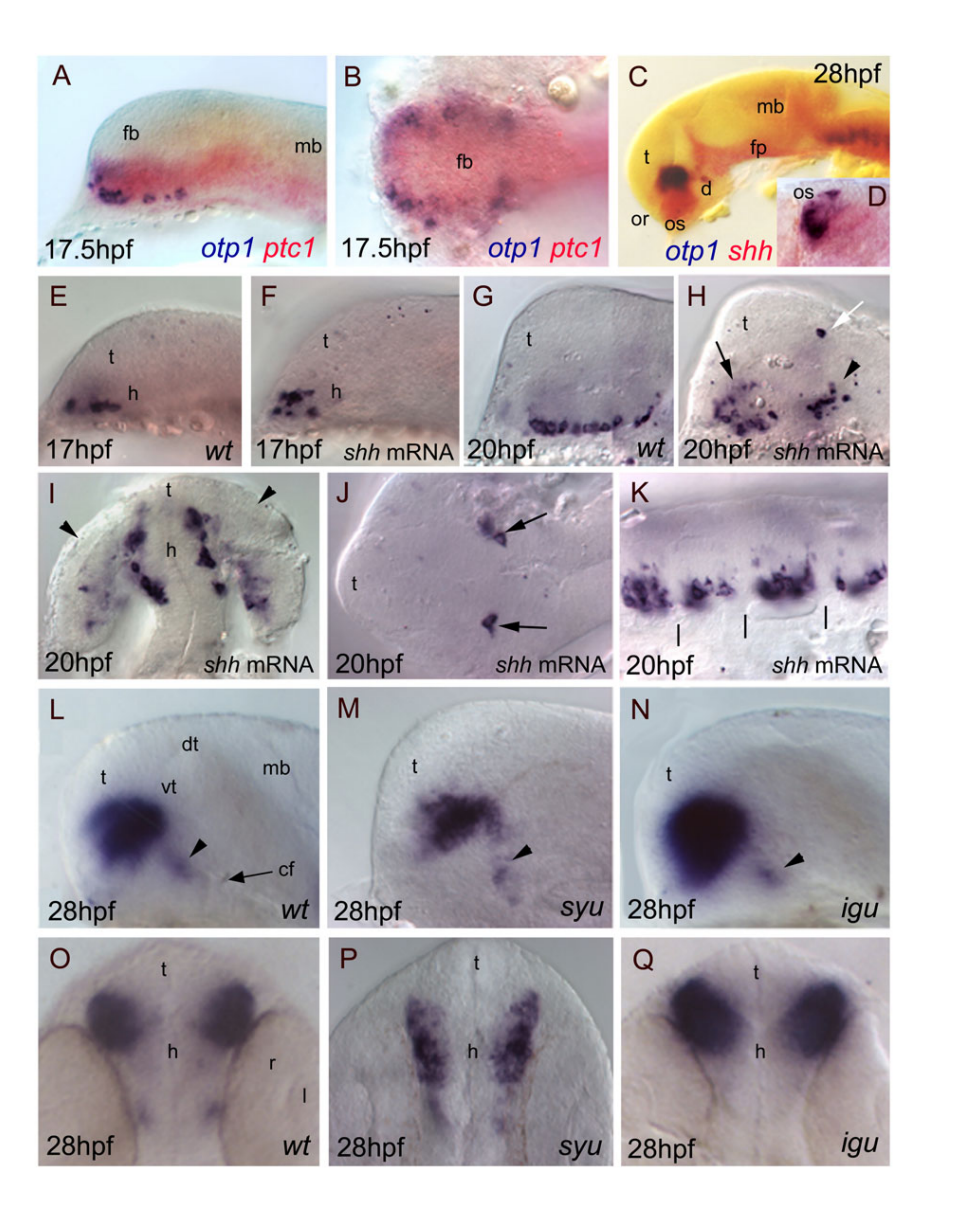
***Relationships between otp1 and HH signaling in the diencephalon***. Anterior is to the left in all panels except for ***D***, ***I***, ***O***-***Q ***where anterior is up. The latter panels, ***B ***and ***J ***are dorsal images, while the remaining ones are lateral views.***A***-***D***, Relative distribution of *otp1 *and *shh *signaling factors, *i.e*. *shh *and its receptor *patched-1 *(*ptc1*). ***A***, ***B***, Co-staining of *ptc1 *(red) and *otp1 *(blue) mRNAs at 17.5 hpf stage reveals that *otp1 *expression is included in the *shh*-pattern, where they are placed at the ventrolateral margins. ***C***, ***D***, Likewise, double labelling of *shh *(red) and *otp1 *(blue) at 28 hpf shows coexpression near the optic stalks, as seen in the close-up (***D***). ***E***-***K***, Overexpression of synthetic *shh *mRNA at 1–2 cell stage induces dorsalization of *otp1 *positive cells. ***E***, ***F***, Since early *otp1 *expression, forced *shh *expression induces an initial dorsal displacement at the rostral edge of the diencephalon, without significant alteration of cell number (17 hpf). ***G***, ***H***, A comparable number of *otp1 *positive cells in both the PO (arrow) and the PT (arrowhead) are dorsalized at later stages of brain development (20 hpf). ***I***, *shh *overexpressing embryos shown in ***H ***display abnormal expression in the optic vesicle (arrowheads). ***J***, In addition, ectopic expression of *otp1 *is consistently found in two symmetrical cells in the dorsal thalamus (open arrow in ***H***, arrows in ***J***). ***K***, The repeated pattern of *otp1 *expression in the hindbrain is maintained, but it now appears as a single median cluster per each rhombomere, and it does not extends dorsally (vertical dashes correspond to rhombomere boundaries; compare with Fig. 4E, F-H). ***L***-***Q***, *otp1 *expression in 28 hpf zebrafish mutants lacking *shh *(*syu*) and the HH-inhibitor *Dzip-1 *(*igu*). Labelling of PO cells indicates that the size of the *otp1 *cluster is (***M***, ***P***) reduced in *syu *and (***N***, ***Q***) enlarged in *igu *as well, if compared with (***L***, ***O***) control embryos. In both mutants, no significant alteration of the *otp1 *cluster in PT is observed (arrowheads in ***L***-***N***). cf, cephalic flexure; d, diencephalon; dt, dorsal thalamus; fb, forebrain; fp, floor plate; mb, midbrain; l, lens; or, optic recess; os, optic stalks; r, retina; t, telencephalon; vt, ventral thalamus.

Searching for regulative interactions between *otp1 *and HH factors, we first compared expression patterns of *shh *and *patched-1 *(*ptc1*), a Shh-specific receptor [38,61], with *otp1*. In the prospective diencephalon, *otp1*-positive cells lie at the ventro-lateral edges of *ptc1 *and *shh *expression domains, therefore in a region exposed to elevate HH concentrations (Fig. 8A–D). Effects of synthetic *shh *mRNA injections on *otp1 *expression were analysed at 17 and 20 hpf of development (Fig. 8E–K). The majority of embryos injected with synthetic *shh *mRNA exhibits a disorganized *otp1 *expression pattern in the hypothalamus. Interestingly, dorsalization of *otp1*-positive cells does not extend to the thalamus, except for two peculiar cells (Fig. 8H,J). Ectopic *otp1 *expression is also observed in the optic vesicle [62] (Fig. 8I). In the hindbrain of *shh*-overexpressing embryos, *otp1 *transcripts are still orderly arranged although as a single domain per rhombomere (compare Fig. 8K with Fig. 4E).

Then, *otp1 *expression was studied in *sonic you *(*syu*) and *iguana *(*igu*) mutant embryos, zebrafish strains characterized by functionally null alleles of, respectively, *shh *[38] and the *dzip1 *gene, a negative regulator of HH signaling gradients [39,40]. In 28 hpf *syu *mutants, the PO *otp1 *pattern is drastically reduced in size (Fig. 8L,M,O,P; Fig. 9A,C). On the contrary, loss of Dzip1 functions in *igu *embryos induces a phenotype characterized by supernumerary PO *otp1*-positive cells (Fig. 8L,N,O,Q). In both mutants, no obvious alterations of *otp1 *expression are observed in the PT (Fig. 8L–Q).

Direct interactions between FGF and HH signaling were approached by WISH analysis of *otp1 *transcripts in *ace*; *syu *double mutant embryos, in which PO or PT clusters of *otp1 *expression are not significantly altered (Fig. 9A,D).

### DA neuronal progenitors and otp1 activity

**Figure 10:**
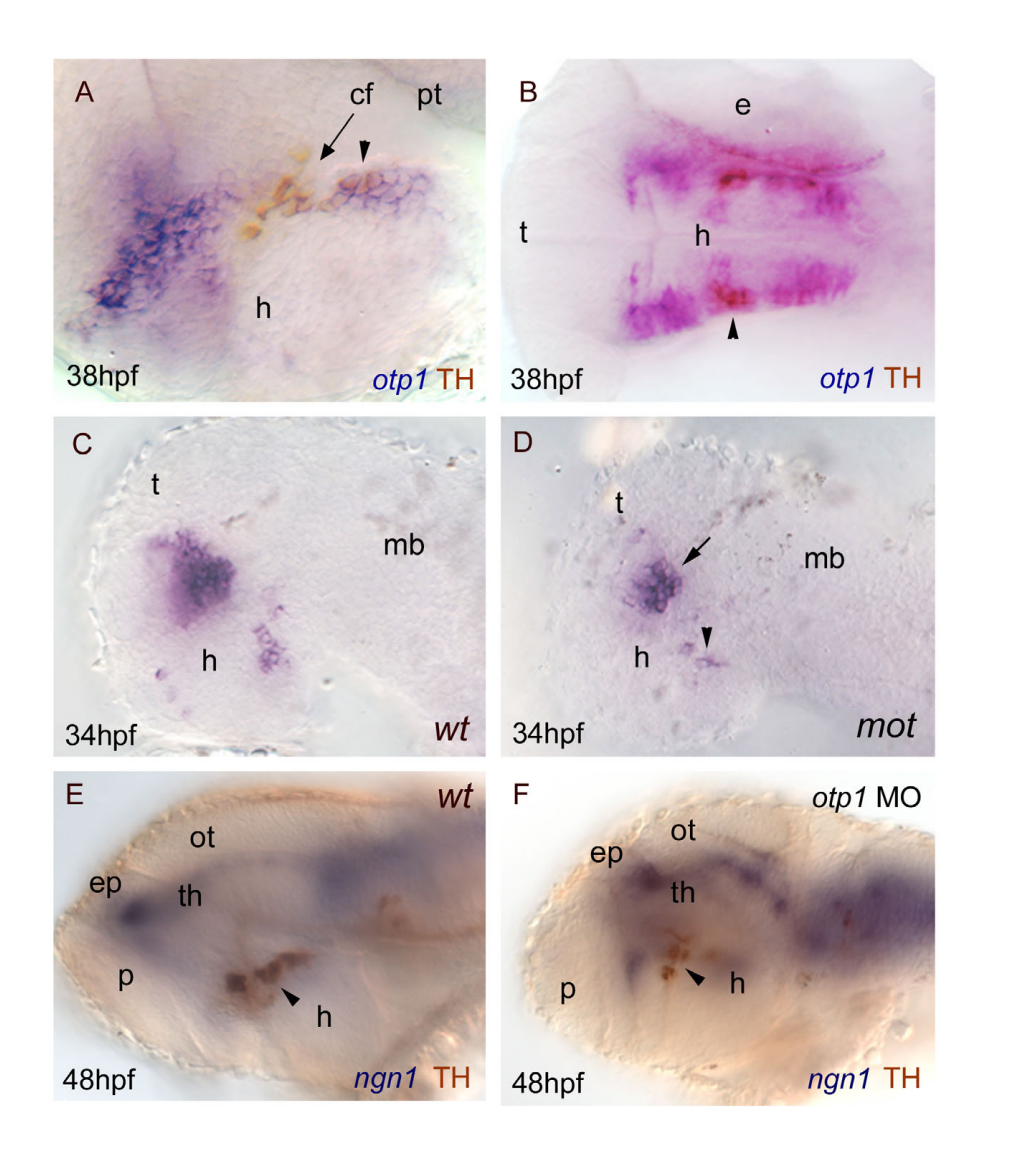
***otp1 functions and CA neuron differentiation***. Anterior is left and dorsal is up (except for ***B***, dorsal view) in all panels. Eyes have been removed. ***A***, ***B***, anti-TH antibody labels the PT diencephalic CA neurons at 38 hpf [65]. Co-labelling with *otp1 *is evident in a fraction of TH-positive neuroblasts in the PT (arrowhead). ***C***, ***D***, *motionless *(*mot*) embryos display a severe reduction of cell numbers across all DA populations of the zebrafish brainstem, including the diencephalic ones (34 hpf) [63]. Herein, absence of *mot *functions drastically inhibits the differentiation of *otp1 *positive cells, more in the PT (arrowhead) then in the PO (arrow). ***E***, ***F***, microinjection of *otp1 *morpholino oligonucleotide lowers the number of TH-labelled DA neuroblasts in the PT (arrowhead) of 48 hpf embryos that were labelled with *neurogenin1 *(*ngn1*), a marker of specific neuronal populations in the forebrain. cf, cephalic flexure; e, eye; ep, epiphysis; h, hypothalamus; mb, midbrain; ot, optic tectum; p, pallium; th, thalamus.

Among populations of CA neurons described in the zebrafish brain during development, the PT hosts distinct groups of DA neuroblasts [63-66]. Our data support the notion that *otp1 *transcription in the PT is functionally linked with a population of DA neurons. Double labelling of *otp1 *mRNA and TH, a marker of CA neurons in the diencephalon, shows that a fraction of the DA neurons placed in the PT, adjacent to the ventral flexure, express *otp1 *(Fig. 10A,B). Moreover, the zebrafish mutant *motionless *(*mot*) features a reduction of DA neurons in the PT [63]. Accordingly, the number of *otp1 *positive cells is sharply decreased in this region in *mot *embryos at 34 hpf (Fig. 10C,D). Finally, inactivation of *otp1 *translation by microinjection of ATG-targeted morpholino oligonucleotides at 1–2 cell stages causes a marked loss of TH-positive cells in the PT (Fig. 10E,F).

## Discussion

*Orthopedia *(*Otp*) expression in vertebrate embryos is crucial for the correct determination of several cell lineages in the neuroendocrine hypothalamus [16,17]. Indeed, *Otp *null-mice die soon after birth due to the developmental failure of the anterior periventricular, paraventricular, and supraoptic hypothalamic nuclei, and the resultant incapability to secrete important neuro-hormones [16,17]. In zebrafish, a detailed characterization of the CA neurotransmitter pathway makes this organism a favourite model system to address the ontogeny of the vertebrate neurosecretory system [63,64,66-77]. To investigate *otp *regulation in vertebrate development, we have isolated two *otp *zebrafish homologues (*otp1 *and *otp2*) and investigated the *in vivo *functions of *shh*, *fgf8*, and *Nodal-related 1 *signaling pathways on *otp1 *expression by means of morpholino-, mRNA- and mutant-based methodologies. The conservative structure of the homeodomain region uncovered between the zebrafish Otp1, Otp2 proteins and the rest of our data set indicates clearly that the above proteins represent members of the Otp gene family of transcription factors. The percentages of identity between the zebrafish Otp homeodomains compared with the ones in *Mus musculus*, *Dugesia japonica *and *Drosophila melanogaster *are found to be variable, reflecting distinct evolutionary relationships as well as functional differences. Moreover, each of the Antp, Otp, and Otd/Otx gene clusters represent phylogenetically distinct clades characterised by elevated levels of sequence and structural homology. Our phylogenetic analysis of Otp proteins highly supports a Vertebrate group, while it highlights two unorthodox subclades, one consisting of Molluscs, Echinoderms and Hemichordates, and one grouping Platyhelminthes, Cnidarians and Arthropods and Tunicates.

*otp1 *gene expression domains are in close proximity to, or within, organizing centres that express signaling factors. In this report, we show that Nodal, SHH and FGF8 independently regulate *otp1 *expression during formation of neural stem cells in the zebrafish forebrain. Since results presented herein centred on functional effects occurring within the first 2 days of development, TH-immunolabeling did not evidence neurons in the PO during our assays, therefore impeding to connect *otp1 *activity with CA neurons in the anterodorsal hypothalamus. However, we report the first case of involvement of *otp *in the determination of a subgroup of ventral hypothalamic CA neurons.

### HH and FGF8 signaling pathways modulate otp1 expression in the hypothalamic preoptic area but not in the posterior tuberculum

HH and FGF8 signaling pathways play key roles in the ventral CNS patterning [78,79]. Our examination of *syu, igu*, and *ace *mutants clearly revealed the dependency of *otp1 *expression on *shh *and *fgf8 *at the level of the *otp1 *positive cluster located in the PO, where the two signaling molecules are synthesized. Absence of Shh protein determines a marked attenuation of the size of the *otp1 *cluster in the PO (alar plate) (Fig. 8L,M,O,P) [37-40]. This result suggests that only the prospective *otp1*-positive cells in the alar plate PO have acquired the competence (*i.e*. additional factors that modulate SHH signaling) to respond to the high SHH concentration from the ventral neural tube and then trigger *otp1 *expression. This is also supported by the evidence that the PO *otp1 *signal increased in *igu *mutants (Fig. 8L,N,O,Q), which are deficient in *shh *negative regulation [39,40]. Inhibition of the SHH pattern via synthetic mRNA microinjection causes profound alterations in the spatial organization of the PO *otp1 *cluster, but does not induce relevant changes in the number of the *otp1*-expressing cells, suggesting that *otp1 *responsiveness in the alar plate is dependent on additional positive regulators rather than negative factors hindering SHH signal propagation.

Interestingly, ectopic *otp1 *expression after *shh *microinjection was selectively observed in the optic placodes (Fig. 8I) and in one fixed pair of cells in the roof of the dorsal thalamus (Fig. 8H,J), suggestive of a predetermination to respond to the *shh *signal.

The *otp1 *signal in the PO appeared significantly expanded in the *ace *mutant (Fig. 9A,B), indicative of a negative role for this signaling factor in the control of PO *otp1 *expression. To confirm this observation we altered the endogenous level of *fgf8 *by synthetic mRNA microinjection: excess of FGF concentration reduces the size of the *otp1*-positive PO cluster. As expected from a potential negative regulator, *fgf8 *microinjection does not induce ectopic expression of *otp1*. No major consequences on *otp1 *expression in the PT are noticed in embryos altered in FGF and SHH signalings (Fig. 8M,P; Fig. 9B,C; data not shown), supporting the conclusion that *fgf8 *and *shh *are not required for *otp1*-based neuronal specification in the posterior diencephalon.

### Nodal affects otp1 expression in the preoptic area and is necessary for otp1 activation in the posterior tuberculum, where otp1 is required for the correct development of catecholaminergic neurons

The *ndr2 *mutant *cyclops *(*cyc*) interferes with the Nodal pathway causing massive defects in the rostral brain [55-58]. Analysis of *otp1 *expression in *cyc *mutant embryos with intermediate phenotypes (reduction of the ventral diencephalon and partial fusion of the eyes) shows midline fusion of PO-specific *otp1 *expression; moreover, we noticed that the domain is decreased in size (compare Fig. 7A,B with Fig. 5G,H). This phenotype could be in part explained by the partial physical deficiency of the PO territory, but also by the repression of *shh *transcription in *cyc *mutants [80], such that the reduction of *otp1*-expressing cells after the disruption of Nodal signaling is determined by intrinsic loss of *shh *(as we observed in *syu *mutants) (Fig. 8M,P), pointing to the PO *otp1 *expression regulation mediated by *ndr2*. We did not detect any *otp1*-expressing cells in the PT of *nodal *(*cyc*) mutant embryos. This phenotype is not caused by *shh *deficiency since *otp1 *expression in the posterior basal plate of the hypothalamus is not modulated by HH signaling, but could be determined by the absence of the prosencephalic ventral structures. This issue has been addressed overexpressing *ndr2 *and analyzing the effects on *otp1 *transcription in the PT area. Increase of Ndr2 function is shown to determine the expansion of the PT-specific *otp1 *domain (Fig. 7C–F), in accordance with the opposite effect in the PT of *cyc *embryos with intermediate phenotypes. Taken together, these results are indicative of Nodal signaling as a positive regulator of *otp1 *expression in the posterior basal plate of the hypothalamus. Furthermore, Holzschuh and colleagues reported the loss of diencephalic DA neurons also in *MZsur*, a Nodal mutant in which the PT correctly develops, therefore suggesting the direct involvement of Nodal in the differentiation of the DA neuron in the posterior tuberculum [81,82].

In this context, our findings allow to propose that the Nodal contribution to the development of the CA neurons in PT might be mediated by *otp1*. Because both *otp1*- and TH-positive neurons occur in the PT, we co-labelled this area. Interestingly, *otp1 *and TH partially overlap in a small clade of neuronal precursors (Fig. 10A,B). In these cells, *otp1 *activation precedes of several hours that of *dopamine transporter *and *tyrosine hydroxylase *genes [64]. In addition, embryos of a zebrafish mutant that displays a reduced number of hypothalamic DA neurons (*motionless, mot*) [63] show less *otp1*-positive neurons (Fig. 10C,D). The hypothesis of a subset of CA neurons requiring *otp1 *action for their differentiation was finally tested by microinjection of *otp1*-specific ATG-targeted morpholino oligonucleotides in wild type embryos. A *mot *mutant phenocopy was generated at the PT level, with the repression of TH-positive cell differentiation (Fig. 10E,F). Altogether, these data confirm the *otp1 *gene as one functional milestone towards the correct development of the CA system.

## Conclusion

**Figure 11:**
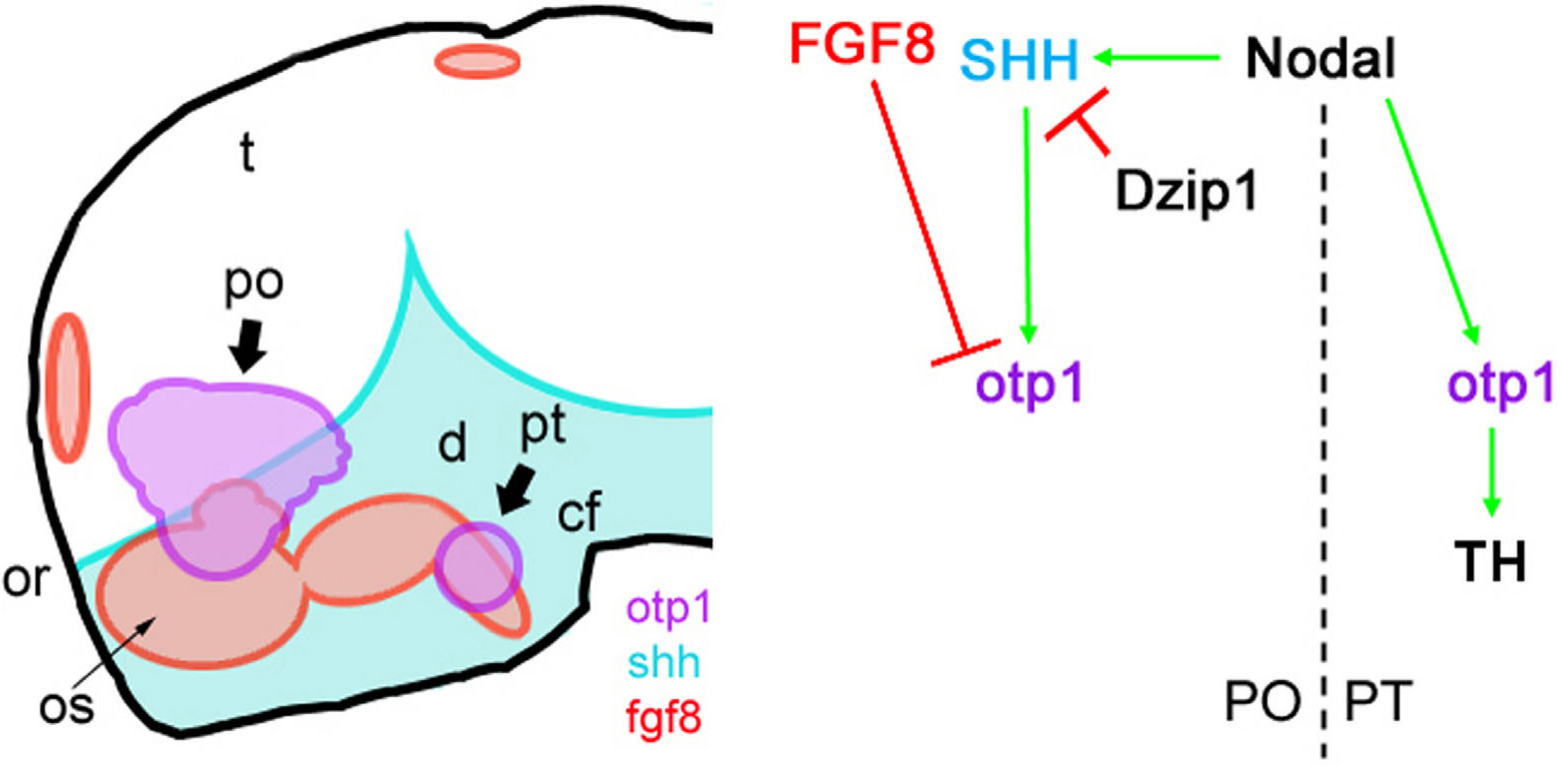
Schematic representation of the spatial and functional links between *otp1 *expression and Nodal, SHH and FGF signaling cascades in the 24 hpf hypothalamic forebrain. ***A***, schematic lateral view of the zebrafish forebrain with anterior to the left and dorsal up. *otp1*, *fgf8 *and *shh *expression domains are painted with different colors (purple, *otp1*; blue, *shh*; red, *fgf8*). ***B***, presumptive Nodal, HH, and FGF signaling cascades genetic interactions regulating *otp1 *expression in the two hypothalamic areas indicated by solid arrows. *otp1 *expression in PO is modulated negatively by FGF8 and positively by SHH; the effect of SHH on *otp1 *expression is diminished by Dzip1, a negative regulator of SHH signaling, and Nodal activates *otp1 *expression through SHH. In PT, *otp1 *expression is independent from HH and FGF signaling cascades, but it is activated by Nodal in a HH-independent way. Nodal-dependent *otp1 *expression in PT is required for the proper differentiation of a subset of CA neurons. cf, cephalic flexure; d, diencephalon; or, optic recess; os, optic stalks; po, preoptic area; pt, posterior tuberculum; t, telencephalon.

In this paper we provide strong circumstantial evidence of regulatory links between *otp1 *expression and Nodal, SHH and FGF signaling cascades in the hypothalamus. The first piece of evidence demonstrates that *Nodal*, in concert with *fgf8 *and *shh*, mediates *otp1 *expression in the preoptic area (Fig. 11). Remarkably, our data indicate a second signaling mechanism in which *nodal*, but not *fgf8 *and *shh*, regulate early *otp1 *functions in the determination of CA neurons in the posterior tuberculum (Fig. 11). The identification of *otp1 *as a key component in the differentiation of diencephalic CA neurons will help in clarifying the developmental bases of several human behavioural aspects as well as pathologies such as addictions and Parkinson's disease.

## Methods

### Animals

Wild type zebrafish of the AB strain were maintained at 28°C on a 14 h light/10 h dark cycle under standard procedures [83]. Embryos were collected by natural spawning, staged according to Kimmel and co-workers [84] and raised at 28°C in fish water (Instant Ocean, 0,1% Methylene Blue) in Petri dishes [85]. We express the embryonic ages in somites (s) and hours post fertilization (hpf). The following mutant alleles were used: *aceti282a *[37], *cycb16 *[55], *mbltm213 *[54], *motm807 *[39,40], *syutbx392 *[38] and *iguts294e *[39,40].

### Cloning

The zebrafish homologue of *orthopedia *(*otp1*) has been isolated by means of PCR from a 16–40 hour old zebrafish embryo cDNA library using degenerate primers ort1_fw (5'-CCNGCNCAGCTSAACGA-3') and ort2_rv (5'-CKYTTYTTCCAYTTNGC-3'), corresponding to PAQLNE and AKWKKR regions of the mouse Otp homeodomain, respectively. The first round of PCR has been carried out using the ort2_rv degenerate primer with the T3 library-vector specific primer. An aliquot of this reaction has been used as template with the ort1_fw and ort2_rv primers. The 150 bp cDNA band obtained encoded a partial homeodomain identical to the corresponding residues of the mouse Otp protein. Gene- and vector-specific primers were used on the same library to isolate the 5' and 3' ends of the *otp1 *mRNA.

### Phylogenetic analysis

A total of 47 sequences, gathered from several EST and genomic databases, were aligned using the ClustalW algorithm [86] as implemented in the Bioedit software v4.7.8 [87] under a variety of gap penalties assigned. Species names, abbreviations, gene names and accession numbers of the sequences are as follows: Bt, *Bos taurus*, Otp XM_604218; Gg, *Gallus gallus*, Otp AY651764; Cf, *Canis familiaris*, Otp XM_546055, Otx2 XM_547830; Pt, *Pan troglodytes*, XM_517691; Hs, *Homo sapiens*, Otp NM_032109, Otx2 NM021728; Mm, *Mus musculus*, Otp CAA71439; Rn, *Rattus norvegicus*, Otp XP_215445; Dr, *Danio rerio*, Otp1 AF071496, Otx1 NM131250, Otx5 NM_181331; Dm, *Drosophila melanogaster*, Otp NM_206187; Pd, *Platynereis dumerilii*, Otx AJ278856; Sp, *Strongylocentrotus purpuratus*, Otp XM_779506, Otx NM_214588; At, *Achaearanea tepidariorum*, Otd AB096074; Tc, *Tribolium castaneum*, Otd1 NM_001039424, Otd2 NM_001039437; Ci, *Ciona intestinalis*, Otp AB210618; Ef, *Euscorpius flavicaudis*, Otd AY738138; Xt, *Xenopus tropicalis*, Otx1 NM_203885; Sk, *Saccoglossus kowalevskii*, Otp AAP79292; Pv, *Patella vulgata*, Otp AF440099; Lv, *Lytechinus variegatus*, Otp AAR17090; Pl, *Paracentrotus lividus*, Otp O76971; Ht, *Heliocidaris tuberculata*, Otp AAS00592; He, *Heliocidaris erythrogramma*, Otp AAS00591; Ds, *Drosophila subobscura*, Antp X60995; Dv, *Drosophila virilis*, Antp AY333070; Am, *Apis mellifera*, Antp NM_001011571; Sc, *Sacculina carcini*, Antp AF393443; Pc, *Podocoryne carnea*, Tbx4/5 AJ581006.

While *Dugesia japonica *(Dj) Otp sequence was obtained directly from Umesono and co-workers [20], *Hydra magnipapillata *(Hm), *Takifugu rubripes *(Tr) and *Tetraodon nigroviridis *(Tn) Otp sequences were generated in the course of this study and are available from the authors. The alignment was manually refined and used as the basis for all tree reconstruction methods. Maximum parsimony (MP) analysis was carried out in PAUP* 4.0b10 (Windows version) [88] using the heuristic search option, 100 random sequence additions and tree bisection-reconnection (TBR) branch swapping. Maximum likelihood (ML) analysis was performed using the quartet puzzling method [89] implemented in the TREE-PUZZLE package (version 5.0) [90] using the heuristic search option and ten random sequence additions. A Bayesian phylogeny was inferred, and posterior probabilities of individual clades were calculated using a variant of the Markov chain Monte Carlo algorithm as employed in MrBayes v3.1.2 [91]. Four Markov chains (three heated, one cold) were run for 200000 generations using random starting trees and the same model employed in branch length estimates, with trees saved every 100 generations. Bootstrap support for individual nodes [92] was calculated on 1000 replicates using the same methods, options and constraints as in the tree-inferences, but removing identical sequences. Maximum Likelihood and Bayesian phylogenetic reconstructions were constrained with the most appropriate model for protein evolution that better fitted the data. Likelihood values for each of 64 candidate models of protein evolution with the best-fitting parameters (gamma distribution, proportion of invariable sites, character frequencies) were computed in the software ProtTest v1.3 [93]. The best aminoacid replacement model for the data was finally calculated using an Akaike (AIC), a second-order Akaike (AICc) and a Bayesian (BIC) information criteria.

### RT-PCR

Total RNAs from 15 samples corresponding to 10 different developmental stage embryos (1–2 cells, 30% epiboly, 50% epiboly, 80% epiboly, 1–2 somites, 8 somites, 10 somites, 15–20 somites, 24 hpf, and 48 hpf) and 3 adult organs (oocyte, brain, testis) were purified, DNase treated, and reverse-transcribed. The cDNAs obtained were tested for the presence of *otp1 *expression using the forward (z*otp*S: 5'-ATGCTCTCTCATGCCGACCT-3') and reverse (z*otp*Rv: 5'-TCTGTTGGTTTTGCTGGCCG-3') primers spanning the cDNA region involved in alternative splicing. The products of the PCRs were loaded and resolved onto 2% agarose gels.

### In situ hybridization and immunohistochemistry

WISH hybridization was carried out according to Thisse and co-workers [47] on embryos fixed for 2 h in 4% paraformaldehyde/phosphate buffered saline, then rinsed with PBS-Tween, dehydrated in 100% methanol and stored at -20°C until processed for WISH [94]. Riboprobes were *in vitro *labelled with digoxigenin or fluorescein (Roche). For double WISH and antibody labelling, WISH was performed first, then embryos were exposed to rat anti-Tyrosine Hydroxilase (TH) (Chemicon) and mouse anti-acetylated α-tubulin (Sigma). Embryos incubated with anti-TH were treated with biotinylated secondary antibody (Vector Laboratories).

### Injections

Synthetic capped *shh*, *ndr2*, and *fgf8 *mRNAs were injected repeatedly (n > 3) at concentrations of 400, 200, and 200 pg per embryo, respectively. Injections were carried out on 1- to 2-cell stage embryos. To repress *otp1 *mRNA translation, an ATG-targeting morpholino was designed (Gene Tools, LLC): 5'-CCAAGAGGTCGGCATGAGAGAGCAT-3'.

## Authors' contributions

LDG and PS designed the study, carried out the wet and *in silico *cloning procedures, the molecular genetic and functional studies, and drafted the manuscript. AP and RT carried out some of the molecular genetic and functional studies. BDB participated to the identification of *otp1 *and preliminary expression analysis. NA carried out sequence alignments and evolutionary analysis. FC participated in the design and coordination of the study. All authors read and approved the final manuscript.
